# Oral Metastasis as a Harbinger of Terminal Lung Adenocarcinoma: A Case Study of Rare and Rapid Decline

**DOI:** 10.7759/cureus.91065

**Published:** 2025-08-26

**Authors:** Shehroz Aslam, Nnenna M Onwumere, Hamzah Akram, Rida Riaz, Aaliya Burza

**Affiliations:** 1 Internal Medicine, Samaritan Medical Center, Watertown, USA; 2 Internal Medicine, Hamilton Health Science, Hamilton, CAN; 3 Family Medicine, Samaritan Medical Center, Watertown, USA; 4 Pulmonary and Critical Care Medicine, Samaritan Medical Center, Watertown, USA

**Keywords:** lung cancer, metastatic lung adenocarcinoma, non-small cell lung cancer (nsclc), oral cavity metastases, oral metastasis

## Abstract

Lung cancer is one of the most prevalent cancers in the United States (U.S), with adenocarcinoma representing the most predominant subtype. Metastasis to the oral cavity from lung adenocarcinoma is exceedingly rare.

This case report describes a patient with lung adenocarcinoma who developed oral metastases, presenting as exophytic lesions along the right maxillary and left mandibular alveolar ridges. The prognostic implications of such metastases are not well-reported in the literature due to the limited number of cases. This case highlights the crucial importance of timely evaluation of oral lesions in patients with known malignancies to facilitate early diagnosis, guide treatment, and improve prognostication and symptom management.

## Introduction

Lung cancer is a leading cause of cancer-related deaths worldwide, with approximately 2.2 million new diagnoses and 1.8 million fatalities reported each year [1]. Non-small cell lung cancer (NSCLC) accounts for more than 85% of all lung cancer cases, with adenocarcinoma being the most common subtype [2]. Adenocarcinomas are known to metastasize to distant organs, including the brain, liver, bones, and adrenal glands. However, oral cavity metastasis is highly uncommon, occurring in fewer than 1% of lung adenocarcinoma cases and usually indicating advanced and aggressive disease [3]. Oral metastases from lung adenocarcinoma often manifest with nonspecific symptoms such as swelling in the gums, bleeding, or dental pain, which may resemble benign dental issues or inflammatory conditions, resulting in potential delays in diagnosis [4]. The occurrence of oral metastasis is associated with a poor prognosis, suggesting significant systemic involvement and limited treatment options [5]. Despite its implications, the prognostic impact of oral metastasis in lung adenocarcinoma is not well understood due to its rarity and the limited number of documented instances.

This case report discusses a 70-year-old male diagnosed with poorly differentiated lung adenocarcinoma, who later presented with bleeding masses in the oral cavity. A biopsy confirmed the metastatic nature of these lesions with an origin of primary lung adenocarcinoma. The rapid and fatal progression of this case highlights the need for early detection and diagnosis of oral metastases in patients with known malignancies such as lung adenocarcinoma. Early identification can help inform prognosis and enable timely intervention.

## Case presentation

**Figure 1:**
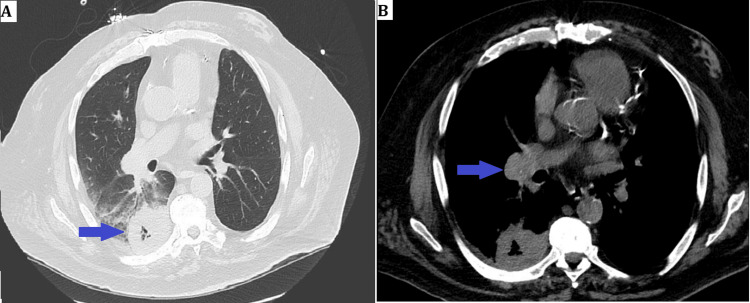
Right lower lobe mass and hilar left anterior descending (LAD) artery A) Right lower lobe necrotic mass measuring 5.1 x 5.5 cm (blue arrow), B) Right hilar lymphadenopathy (blue arrow)

A 70-year-old male with a recently identified lung mass presented to our facility following several weeks of worsening hemoptysis and cough. Initially, he experienced intermittent cough and minimal hemoptysis for about two weeks. His primary care physician had performed a non-contrast chest CT (Figure 1), which revealed a necrotic mass in the right lower lobe measuring 5.1 x 5.5 cm, alongside mediastinal and right hilar lymphadenopathy (maximum size: 3 cm in the right inferior hilar region). The worsening of his symptoms led to his hospitalization.

Upon admission, the patient reported generalized weakness, decreased appetite, and approximately 10 pounds of unintended weight loss over the previous weeks. His vital signs were stable, but laboratory tests indicated anemia with a hemoglobin level of 7.8 g/dL (reference range 13.5-17.5 g/dL), an elevated white blood cell count of 12,900/µL (reference range 4000-10000/µL), and stage III chronic kidney disease, with a stable creatinine level of 1.6 mg/dL (reference range 0.70-1.30 mg/dL). Social history was notable for a 10-pack-year smoking history (with cessation at age 50) and a significant family history of lung cancer in two siblings.

**Figure 2:**
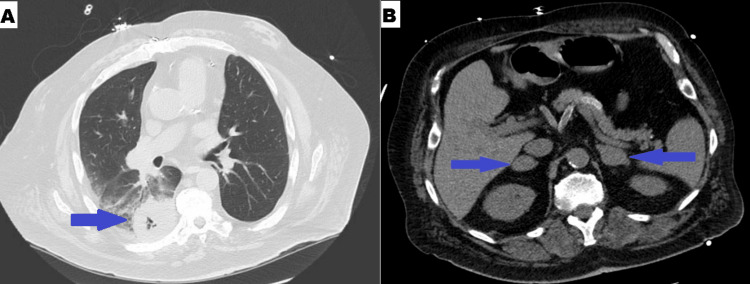
CT scan showing right lung and adrenal masses A) Right lower lobe necrotic mass (blue arrow), B) Right and left adrenal masses (blue arrows)

A follow-up CT scan during the admission showed a similar-appearing right lower lobe cavitary mass, along with ongoing lymphadenopathy. Additionally, CT abdomen and pelvis revealed bilateral adrenal masses concerning for metastases (the largest measuring 3.1 cm on the left) (Figure 2).

**Figure 3:**
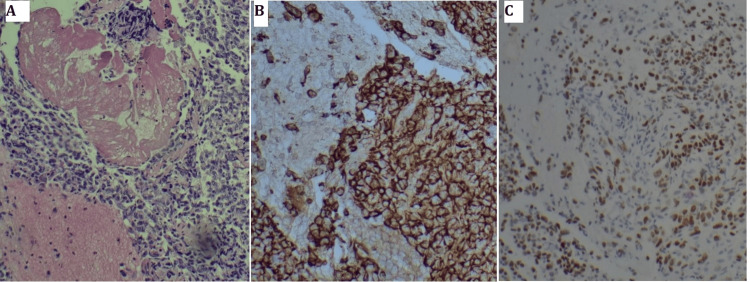
Right hilar lymph node histopathology showing positivity for MCK and TTF-1 A) Right hilar lymph node (HE, x40), B) Right hilar lymph node positive for MCK stain, C) Right hilar lymph node positive for TTF-1

The patient received two units of packed red blood cells for symptomatic anemia. He underwent inpatient bronchoscopy with endobronchial ultrasound (EBUS), which showed no active bleeding or visible endobronchial lesions. Transbronchial needle aspiration (TBNA) from the right hilar lymph nodes (R4, R10) confirmed poorly differentiated lung adenocarcinoma, with immunohistochemistry positive for TTF-1, MCK, and focal Ber-EP4, and negative for p63, p40, CK5/6, napsin, CD56, and synaptophysin (Figure 3).

**Figure 4:**
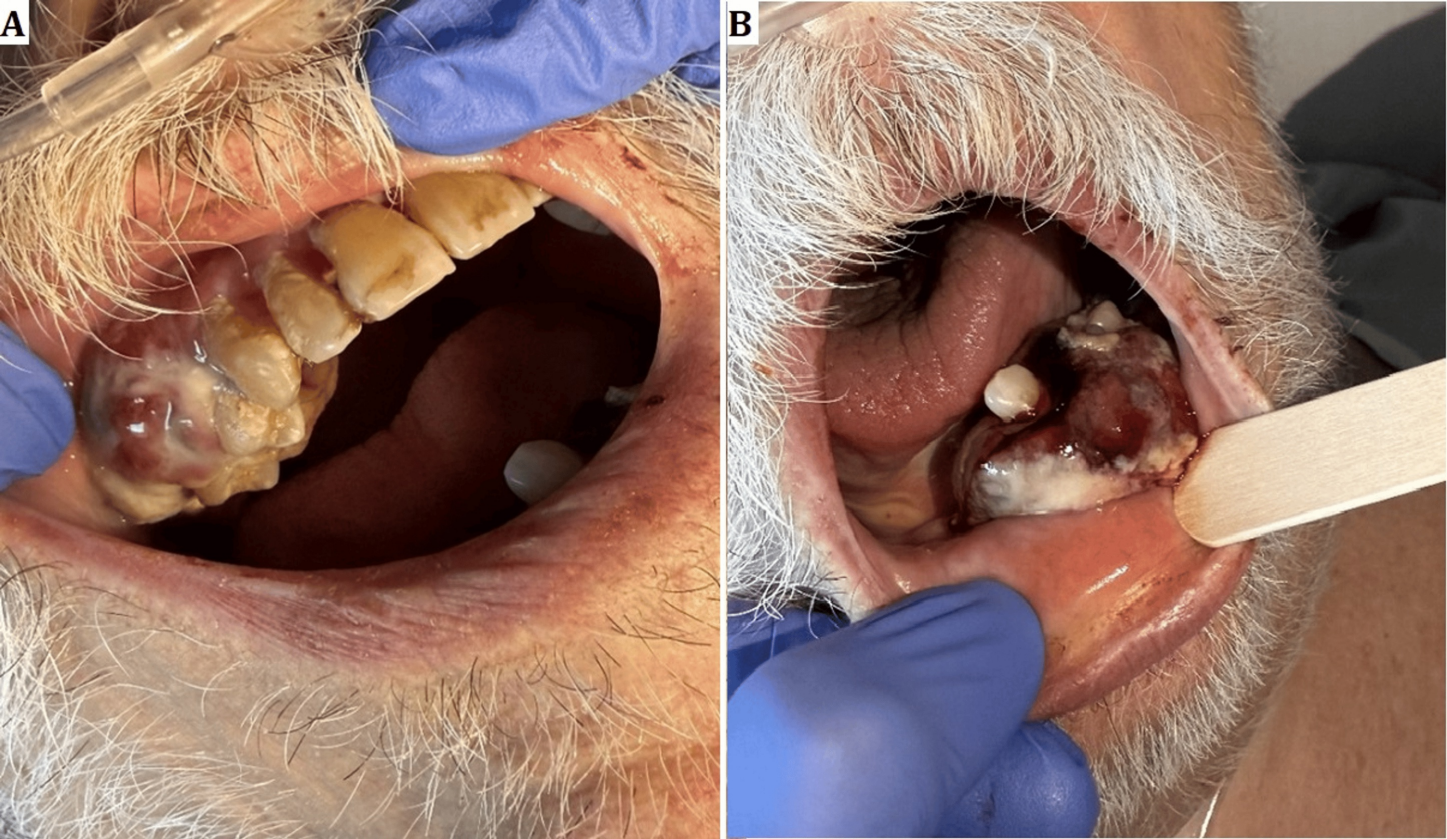
Bleeding mass in the maxillomandibular alveolar ridges. A) Right maxillary alveolar ridge mass, B) Left mandibular alveolar ridge mass

While hospitalized, the patient developed gum bleeding localized to the right maxillary alveolar region and swelling of the left mandible. Physical examination revealed two exophytic oral masses: one in the right maxillary alveolar ridge and the other in the left mandibular alveolar ridge, both with active bleeding (Figure 4).

**Figure 5:**
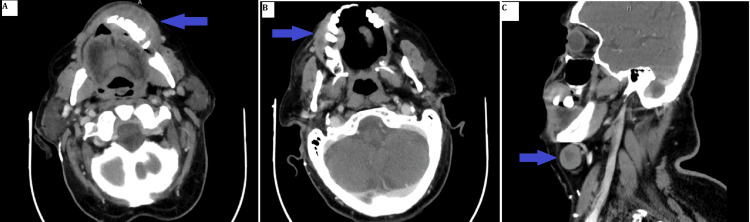
CT images showing alveolar masses and submental LN A) Left lower alveolus mass (blue arrow), B) Right upper alveolus mass (blue arrow), C) Right submental lymph node (blue arrow)

**Figure 6:**
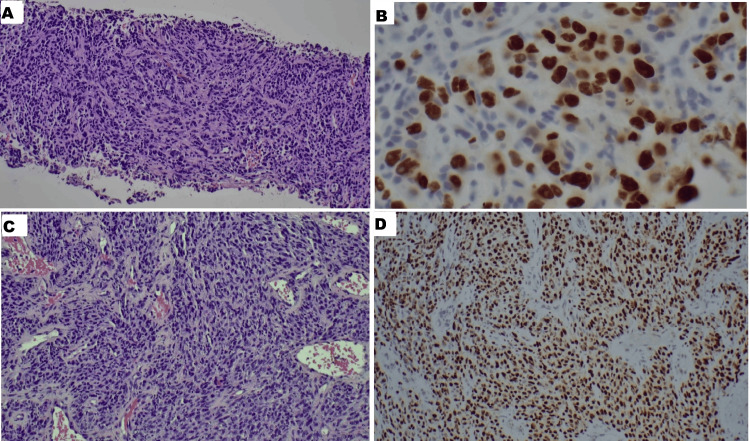
Histopathology of the submental lymph node and alveolus mass A) Right submental lymph node HE, x 40, B) Right submental lymph node showing TTF1 positivity, C) Left lower alveolus mass HE, x 40, D) Left lower alveolus mass showing TTF1 positivity

Maxillofacial and neck CT scans revealed a 5.2 × 1.8 cm soft tissue lesion over the left anterior mandible, suspicious for an inflammatory mass versus neoplasm. Additional findings included a 2 × 3 cm soft tissue swelling over the right anterior maxillary bone, accompanied by bulky submental lymphadenopathy (right greater than left) (Figure 5). Ultrasound-guided fine needle aspiration (FNA) of the submental nodes and punch biopsies of the alveolar lesions confirmed metastatic lung adenocarcinoma, which was diffusely positive for TTF-1 and negative for P40 and P63 (Figure 6).

The patient was evaluated by both the radiation oncology and otolaryngology teams. Given the resolution of bleeding and improvement in symptoms, radiation therapy for the oral metastases was planned in the outpatient setting. Systemic treatment was deferred pending the results of next-generation sequencing, and the patient was discharged with close outpatient follow-up with radiation and medical oncology.

Unfortunately, within a week, he was readmitted due to sepsis, acute respiratory failure attributed to post-obstructive pneumonia, pulmonary embolism, and gastrointestinal bleeding. Despite aggressive measures, including endoscopy and mesenteric embolization, his condition deteriorated, leading to multiorgan failure. The patient transitioned to comfort-focused measures and passed away shortly thereafter.

Next-generation sequencing revealed high PDL1 expression (tumor proportion score of 80%) and the absence of EGFR and BRAF mutations, as well as ALK fusions, indicating that immunotherapy could have been a viable systemic treatment option (Table 1). However, the rapid progression of his cancer and associated complications precluded this approach.

**Table 1:** NGS results showing tested markers and viable treatment options Abbreviations: FDA (Food and Drug Administration), NCCN (National Comprehensive Cancer Network), NGS (Next Generation Sequencing), PD-L1 IHC (Programmed Death-Ligand 1 Immunohistochemistry), ALK (Anaplastic Lymphoma Kinase), EGFR (Epidermal Growth Factor Receptor), NTRK (Neurotrophic Receptor Tyrosine Kinase)

Genomic Variant Clinical Significance Classification			
Clinical benefits in this patient’s tumor type			
Tumor Markers	Treatment Regimen	Line of Treatment	Sources
Positive: PD-L1 IHC (22C3)	Cemipilmab, pembrolizumab	First line	FDA (approved) NCCN
-	Pembrolizumab	Subsequent line	FDA (approved) NCCN
Negative: ALK fusion, EGFR	Atezolizumab + bevacizumab + carboplatin + paciltaxel, atezolizumab + carboplatin + nab-paclitaxel, ipilimumab+ nivolumab+ platinum doublet, pembrolizumab+pemetrexed+platinum chemotherapy	First line	FDA (approved) NCCN
-	Durvalumab + tremelimumab + platinum chemotherapy	Metastatic	FDA (approved) NCCN
Negative: ALK fusion, EGFR, ROS1 fusion	Cemiplimab + platinum chemotherapy	First line	FDA (approved) NCCN
Negative: ALK fusion, BRAF, V600E, EFGR, ERBB2 mutation, KRAS G12C, MET Exon 14 skipping, NTRK1 fusion, NTRK2 fusion, NTRK3 fusion, RET fusion, ROS1 fusion	Pembrolizumab + carboplatin + (paclitaxel or nab-paclitaxel)	First line	NCCN

## Discussion

This case emphasizes the notably aggressive clinical course and poor prognosis associated with oral metastases from lung adenocarcinoma, a highly uncommon presentation with limited documentation in existing literature. Our patient's rapid clinical decline, from initial diagnosis to death within a matter of weeks, is consistent with previous reports highlighting oral metastases as indicators of advanced disease stage and significantly shortened survival [6]. Metastasis, a hallmark of malignancy, is characterized by the widespread dissemination of tumor cells; however, only a fraction of these cells successfully colonize distant sites [7]. Oral cavity metastases from lung adenocarcinoma are exceedingly rare, accounting for less than 1% of lung cancer cases [7]. However, the actual frequency may be underestimated, as noted by Meyer and Shklar, due to the rarity of maxillary bone examinations during autopsies [8].

Hirshberg et al. explored the uncommon nature of oral metastases in their review of 673 oral malignancies, identifying only a limited number of cases affecting the jawbones and oral mucosa [9]. Seoane et al. further characterized this rarity, noting a predominance in male patients around the sixth decade of life and typically occurring in individuals with previously diagnosed primary malignancies [10]. These demographic trends were confirmed by Wendling et al., who observed a marked male predominance (33 males vs. 13 females) and frequent mandibular involvement in oral and maxillofacial metastasis of lung cancer [11]. Clinically, a higher incidence of oral adenocarcinoma metastasis is observed in the mandible (55-80% of cases) compared to the maxilla (approximately 25% of cases), which may be attributed to differences in vascular supply within the tissues [12]. Our patient, however, had synchronous involvement of both maxillary and mandibular bone as noted in the right upper alveolus and left lower alveolus, respectively (Figures 4-5).

Reports in the literature highlight tongue metastasis as a rare but documented manifestation of lung cancer. Zegarelli et al. identified 2 cases among over 6,800 autopsies, Baden et al. reported a 21% incidence localized to the base of the tongue, and Cheng et al. also described similar cases [13-15].

Clinically, oral metastases often present with symptoms such as pain, swelling, bleeding, loosening or displacement of teeth (28.6%), and paresthesia (30%) [16]. Rajappa et al. described oral metastatic tumors as aggressively growing lesions associated with severe morbidity, including dysphagia, chewing difficulties, disfigurement, and hemorrhage, all of which were observed in our patient [17]. Diagnostically, the nonspecific and varied clinical presentations of oral metastases pose significant challenges, frequently mimicking benign conditions, necessitating a high index of clinical suspicion and timely biopsy for accurate diagnosis [18]. Immunohistochemically, thyroid transcription factor-1 (TTF-1) positivity, demonstrating approximately 85% specificity for lung primaries, is a critical marker for distinguishing metastatic lung adenocarcinoma [19].

Patients diagnosed with oral metastases face an exceedingly poor prognosis, typically experiencing a median survival of only three to six months due to rapid progression and extensive metastatic involvement [20]. Our patient's swift deterioration, occurring within weeks of oral lesion detection, aligns closely with the dire outcomes reported in the literature. Emerging evidence suggests that oral metastatic involvement represents an especially aggressive disease phenotype, characterized by rapid dissemination, with approximately 80% of affected patients developing further metastases within 1 month and 60% succumbing within 8 weeks [20]. This case reinforces the importance of early recognition, prompt diagnostic intervention, and symptom-focused management strategies in patients presenting with oral lesions and a known diagnosis of malignancy.

## Conclusions

This case of lung adenocarcinoma metastasizing to the oral cavity underscores the poor prognosis associated with such presentations and highlights the need for clinical suspicion, timely diagnosis, and appropriate palliative interventions. In our patient, the development of exophytic, bleeding oral masses coincided with rapidly worsening systemic symptoms and a marked decline, signifying advanced, aggressive disease. While hemoptysis is often attributed to primary pulmonary pathology, concurrent bleeding from oral metastases may complicate the clinical picture, emphasizing the importance of thorough physical examination and prompt tissue diagnosis. The rapid progression observed here is consistent with prior reports describing shortened survival and aggressive courses in patients with oral metastases, reinforcing the need for vigilance and early detection. Furthermore, this case supports the broader perspective that oral metastases represent a clinical marker of disseminated disease and poor prognosis, aligning with emerging views that such manifestations indicate an aggressive phenotype.
